# Runcaciguat, a novel soluble guanylate cyclase activator, shows renoprotection in hypertensive, diabetic, and metabolic preclinical models of chronic kidney disease

**DOI:** 10.1007/s00210-021-02149-4

**Published:** 2021-09-22

**Authors:** Agnès Bénardeau, Antje Kahnert, Tibor Schomber, Jutta Meyer, Mira Pavkovic, Axel Kretschmer, Bettina Lawrenz, Elke Hartmann, Ilka Mathar, Joerg Hueser, Jan R. Kraehling, Frank Eitner, Michael G. Hahn, Johannes-Peter Stasch, Peter Sandner

**Affiliations:** 1 Cardiovascular Research, Pharma Research Center, Bayer AG, Aprather Weg 18A, 42096 Wuppertal, Germany; 2 Novo Nordisk, Bagsværd, Denmark; 3 Division of Nephrology and Clinical Immunology, RWTH Aachen University, 52062 Aachen, Germany; 4 Institute of Pharmacy, Martin Luther University, 06120 Halle, Germany; 5 Institute of Pharmacology, Hannover Medical School, 30625 Hannover, Germany

**Keywords:** cGMP, sGC activator, Runcaciguat, CKD, DKD

## Abstract

Chronic kidney diseaQueryse (CKD) is associated with oxidative stress which can interrupt the nitric oxide (NO)/soluble guanylyl cyclase (sGC) signaling and decrease cyclic guanosine monophosphate (cGMP) production. Low cGMP concentrations can cause kidney damage and progression of CKD. The novel sGC activator runcaciguat targets the oxidized and heme-free form of sGC, restoring cGMP production under oxidative stress. The purpose of this study is to investigate if runcaciguat could provide an effective treatment for CKD. Runcaciguat was used for the treatment not only in rat CKD models with different etiologies and comorbidities, namely of hypertensive rats, the renin transgenic (RenTG) rat, and angiotensin-supplemented (ANG-SD) rat, but also in rats with diabetic and metabolic CKD, the Zucker diabetic fatty (ZDF) rat. The treatment duration was 2 to 42 weeks and runcaciguat was applied orally in doses from 1 to 10 mg/kg/bid. In these different rat CKD models, runcaciguat significantly reduced proteinuria (urinary protein to creatinine ratio; uPCR). These effects were also significant at doses which did not or only moderately decrease systemic blood pressure. Moreover, runcaciguat significantly decreased kidney injury biomarkers and attenuated morphological kidney damages. In RenTG rats, runcaciguat improved survival rates and markers of heart injury. These data demonstrate that the sGC activator runcaciguat exhibits cardio-renal protection at doses which did not reduce blood pressure and was effective in hypertensive as well as diabetic and metabolic CKD models. These data, therefore, suggest that runcaciguat, with its specific mode of action, represents an efficient treatment approach for CKD and associated CV diseases.

**Graphical abstract:**

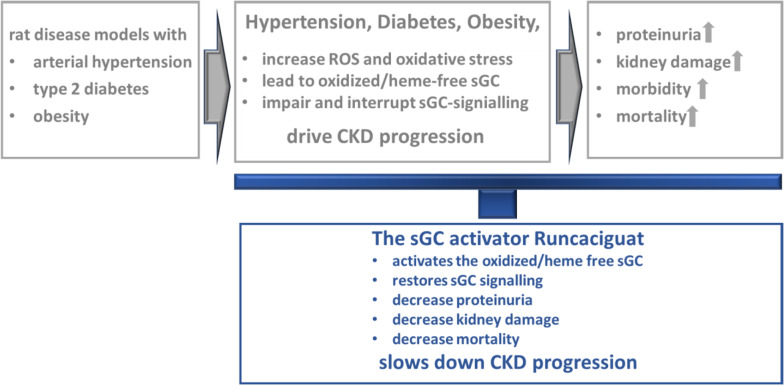

**Supplementary Information:**

The online version contains supplementary material available at 10.1007/s00210-021-02149-4.

## Introduction

The nitric oxide (NO), soluble guanylyl cyclase (sGC), cyclic guanosine 3′,5′-monophosphate (cGMP) signaling cascade (NO-sGC-cGMP pathway), is a pivotal signaling pathway that regulates many cells, tissues, and organ functions. Dysregulation and reduced formation of the second messenger cGMP plays an important role in cardiovascular and cardiopulmonary diseases such as chronic heart failure and pulmonary hypertension (Stasch et al., 2011; Pieske et al., 2014; Gheorghiade et al., 2015). It has also been shown that cGMP is a prominent regulator of kidney function and could be involved in the regulation of not only cortical renal blood flow of afferent and efferent arterioles but also of medullary perfusion. In addition, cGMP could impact not only renin secretion but also tubular transport mechanism (Krishnan et al., 2018, Carlstrom 2021).

Common comorbidities in cardiovascular and kidney disease like hypertension, diabetes, or obesity are leading to endothelial dysfunction and the impairment of cGMP production which can not only cause a progressive damage of blood vessels but also trigger end-organ damage in the heart and the kidney. This ultimately can not only result in chronic kidney disease (CKD) and end-stage renal disease (ESRD) but also chronic heart failure (HF). Therefore, restoring cGMP signaling could become a powerful treatment option for cardiac and renal damage of different etiologies (Salloum et al., 2012; Stasch et al., 2015; Friebe et al., 2019; Follmann et al., 2013; Sandner et al., 2021). However, comorbidities like hypertension, diabetes, and obesity are causing high and persistent levels of oxidative stress. The molecular pathophysiology caused by oxidative stress is at least in part driven by oxidation of the ferrous heme bound to the sGC from Fe^2+^ to Fe^3+^ which destabilizes the heme group and subsequently leading to the heme-free sGC which cannot bind NO. Therefore, oxidative stress interrupts and impairs NO signaling, by NO unresponsiveness of the sGC, causing endothelial dysfunction and cardiovascular and cardio-renal diseases (Gladwin, 2006). Thus, treatment approaches stimulating NO production (nitrates, NO donors) or inhibiting cGMP degradation (PDE5, PDE9 inhibitors) are of limited efficacy caused by impaired NO binding to the sGC and the low endogenous cGMP levels. The discovery of NO-independent sGC stimulators and sGC activators could overcome these limitations of NO donors and PDE inhibitors and can target sGC directly. Thus, sGC stimulators and sGC activators hold big promise for the treatment of cardiovascular diseases. In fact, the first in class sGC stimulator riociguat was approved in 2013 for the treatment of pulmonary arterial hypertension (PAH) and chronic thromboembolic pulmonary hypertension (CTEPH) (Ghofrani et al., 2017) and vericiguat completed successfully a pivotal phase 3 clinical trial in HF patients (Armstrong et al., 2020b) and was recently approved for HF treatment. In contrast to sGC stimulators, sGC activators can bind and activate the oxidized and heme-free sGC and, therefore, sGC activators may especially be useful under persistent oxidative stress condition. As pointed out before, this may be the case in CKD and ESRD as the kidney is particularly vulnerable to oxidative stress. Hence, sGC activators could provide effective treatment options in renal diseases.

Bayer has discovered the novel, oral sGC activator runcaciguat (BAY 1,101,042) which is selectively binding oxidized and heme-free sGC leading to a concentration-dependent increase in cGMP production in vitro, resulting in blood vessel relaxation ex vivo, and blood pressure reduction in vivo with a favorable once-daily pharmacokinetic (PK) profile (Hahn et al., 2021). Given the unique profile of runcaciguat and the treatment potential of sGC activators in CKD, we were aiming to broadly investigate the renoprotective effects of runcaciguat in vivo, in three rodent models of CKD with different etiologies and comorbidities. To this end, runcaciguat was given as a chronic oral treatment to (a) a hypertensive model of CKD in angiotensin II-treated Sprague–Dawley rats (ANG-SD), (b) a hypertensive CKD model, the renin transgenic (RenTG) rat [TGR(mRenR2)27] (Mullins et al., 1990, Ganten et al., 1991), with further increased oxidative stress burden and endothelial dysfunction by chronically supplemented NO synthase inhibitor L-NAME, and (c) obese and diabetic Zucker diabetic fatty (ZDF) rats [(ZDF-Lebr^fa^-Crl)] also characterized by progressive loss of kidney function (Shiota and Printz, 2012). Our results suggest that the novel NO- and heme-independent sGC activator runcaciguat could significantly attenuate or even stop further renal deterioration in these models and leads to a reduced overall mortality. Therefore, runcaciguat may become an effective treatment option for prevention of CKD associated with hypertension, diabetes, and obesity.

## Material and methods

### Compounds

The sGC activator runcaciguat (BAY 1101042), was synthetized at Bayer AG, Wuppertal, Germany (Hahn et al. 2021). Runcaciguat was either dissolved in vehicle (transcutol, 20% cremophor, and 70% water (Ren2TG study and Ang-SD study)) and prepared freshly before administration or was mixed in the food (ZDF study).

### Animal models

Animal experiments were conducted by Bayer AG (Wuppertal) in accordance to the current national legislation (German protection of animals’ act and the EU directives on the protection of animals used for scientific purposes). Study protocols were approved by the regional regulatory authority (LANUV NRW in Germany) and by the institutional animal care and use committee of Bayer AG. Organ harvesting, histological examination, urine and blood collection, gene expression, pharmacokinetics methods, invasive, or non-invasive blood pressure measurements (tail-cuff) are detailed in the other paragraphs, below.

#### ANG-SD rat model

Experimentally, chronic renal failure in male Sprague–Dawley (SD) rats was induced by angiotensin II (ANG II) infusion (450 ng/min/kg, Merck, Calbiochem®), via subcutaneous osmotic minipumps for 14 days (Alzet model 2002). Under ANG II infusion, SD rats develop progressive kidney structural alterations (interstitial fibrosis and glomerulosclerosis) and kidney functional changes (polyuria, proteinuria, and an impaired glomerular filtration rate). In this study, male SD rats (Harlan) were weighing ca. 260 g before the pump implantation, corresponding to approximately 12 weeks of age. The minipumps were preincubated in vitro in 0.9% saline at 37 °C for 14 h before surgical implementation. Pumps were placed into the subcutaneous space of isoflurane anesthetized rats through a small incision in the flank. Treatment started 1 day after implantation of the osmotic minipump. Before treatment, rats were randomized into five groups (*n* = 12/group) according to body weight and were treated orally, per oral gavage, either with placebo (vehicle, 2 ml/kg, once daily) and runcaciguat given per oral gavage (0.3, 1.0, and 3.0 mg/kg/bid) for a total of 13 consecutive days. In parallel, naïve SD rats served as sham (control) and were treated with vehicle. The body weight of all animals was recorded daily (File Nr WDPOS11/21).

#### RenTG rat model

Seventy eight (78) male renin transgenic [TG(mRRen2)27] (RenTG) rats of 8 weeks of age were used. Rats were randomized in 4 groups with 24 in group 1, 18 rats in group 2, 18 rats in group 3, and 18 rats in group 4. All groups were then chronically supplemented with L-NAME administrated via drinking water (30–50 mg/l). All groups were treated twice daily (bid) per oral gavage with either placebo/vehicle consisting of 10% transcutol, 20% cremophor, and 70% tap water (group 1) or vehicle + 1, + 3, and + 10 mg/kg/bid runcaciguat in group 2, group 3, and group 4, respectively. Study duration with concomitant NO synthase inhibitor N-nitro-L-arginine methyl ester (L-NAME) administration and bid treatment was 8 weeks. Mortality and body weight were assessed daily (File Nr PH40762).

#### ZDF rat model

Forty (40) male diabetic rats ZDF [(ZDF-Lebr^fa^-Crl) (Charles River Germany)] at the age of 22 weeks were randomized into two groups and received either runcaciguat with 140 ppm mixed in standard rat chow (*n* = 20 rats) or standard rat chow only (control group, *n* = 20 rats). Treatment duration given as food admix was 42 weeks. The dose of 140 ppm was chosen to fit the plasma exposure achieved after a pre-study performed in ZDF rats with 3 mg/kg/bid oral treatment per oral gavage over 7 weeks (in-house data; Table S1). Consequently, runcaciguat amount per food was calculated based on the ZDF food intake. Body weight was assessed every 3 weeks and survival was calculated daily (File Nr PH40763).

### Blood pressure measurements

A micro-tip pressure transducer catheter (Millar Instruments) was inserted to isoflurane-anesthetized (2%) ANG-SD rats on day 14 of the study, in order to record hemodynamic parameters. The catheter was implanted via the right carotid artery, then quickly advanced into the left ventricle to allow the measurement of systemic blood pressure in a short time (2 min). In Ren2TG and ZDF rats, blood pressure was measured in conscious conditions via tail-cuff method before the start of the study (day 0) and on regular interval until the end of the studies.

### Biomarker measurements

#### Urine and plasma

Urine collection was performed at baseline and regularly during the course of the studies. Rats were placed in metabolic cages diuresis for 6–8 h. Urine volume, protein, urea, and creatinine contents were measured. At necropsy, blood was collected on EDTA, then processed to plasma that was stored at − 20 °C until analyzed. Plasma and urine were collected for measurement of kidney and heart biomarkers by ELISAs. All assays were performed according to manufacturers’ instructions: OPN, Mouse/Rat Osteopontin Quantikine ELISA Kit (R&D); cystatin C and NGAL ELISAs (BioVendor Laboratory Medicine, Inc.); KIM-1 ELISA (Bioassay Works); and H-FABP, Rat H-FABP ELISA Kit (Hycult Biotech).

#### Gene expression in kidneys

For semiquantitative gene expression profiling, total RNA from kidney tissue was isolated using Trizol (Invitrogen, Thermo Fisher Scientific). RNA was reversely transcribed into cDNA with the ImProm II Reverse Transcription System (Promega) according to the manufacturer’s instructions. Specific primers and FAM/TAMRA probes were designed for genes of interest and qPCR was performed with the qPCR Master Mix Plus (Eurogentech; Seraing, Belgium) on a 7900 HT Fast Real-Time PCR System (Applied Biosystems, Thermo Fisher Scientific). Ct values were determined by Applied Biosystems’s SDS Software (version 2.4), normalized to the housekeeping gene Rpl32.

### Pharmacokinetic measurements

The pharmacokinetic properties (PK) of runcaciguat were assessed after chronic administration in rats. Blood samples were collected from a jugular or tail vein (tail vein preferred for PK samples). Approximately 0.1 ml of whole blood was collected into blood tubes containing K3-EDTA anticoagulant at each timepoint. Samples were processed to plasma and the harvested plasma was stored at − 80 °C until further analysis. Plasma concentrations of runcaciguat were determined after protein precipitation with acetonitrile (ACN) containing an internal standard using a qualified high-performance liquid chromatography hyphenated to tandem mass spectrometry (LC–MS/MS) method. As an internal standard (ISTD), a stable isotope labeled (SIL) standard ([2H9] runcaciguat) was prepared inhouse. All measurements were performed on an Agilent 1290 Infinity system (Waldbronn, Germany) hyphenated to an API 5000 Triple Quadrupole mass spectrometer (Sciex, Canada). The chromatographic separation was performed on an YMC Meteoric Core C18 column (100 × 2.1 mm, 2.7 µm). For temperature control, the column oven was set to a temperature of 40 °C. The flow rate was adjusted to 0.35 ml min^−1^ using an injection volume of 5 μL. The mobile phases consist of 2 mM ammonia acetate buffer (AAc) adjusted to pH 3 (mobile phase A) using formic acid (FA) and acetonitrile (ACN, mobile phase B) as organic solvent. The separation was performed under isocratic conditions at 60%B for 4 min. Afterwards, the amount of mobile phase B was increased to 95%B to remove remaining hydrophobic interferences/contaminants from the system before flushing the system back to the initial mobile phase composition for the next analysis. For MS data acquisition, the multiple reaction monitoring mode (MRM) was applied using positive electrospray ionization (ESI). The monitored mass transitions (m/z, mass to charge ratio) for runcaciguat and corresponding ISTD were m/z 488.2 to m/z 222.2 for runcaciguat and m/z 497.1 to m/z 227.0. The MS ion source temperature was set to 450 °C and ionization voltage was 4.5 kV. Nebulizer and drying gas were both set to 35 psi and collision activated dissociation gas (CAD) was adjusted to 4. The calibration range in plasma ranged from 1.00 to 200 mg/L. In all analytical runs, quality control (QC) samples were prepared and analyzed. For data acquisition and analysis, Analyst 1.6.2 and the internal LIMS were used.

### Necropsy and histopathology

At the end of each study, animals were kept in depth narcotic (isoflurane, 5–10%) and bled via aortic puncture and sacrificed by permanent inhaled isoflurane, and blood was taken in order to measure plasma pharmacodynamic parameters. Heart and kidney were harvested, weighed, rinsed, and then fixed for histological evaluation or immediately frozen for analysis of expression of kidney damage marker genes. Kidney samples were fixed in Davidson’s solution and embedded in paraffin. Paraffin sections were prepared and stained with hematoxylin and eosin. The slides were analyzed using a semiquantitative scoring, ranging from grade 1 to 5 (grade 1, minimal/very few; grade 2, slight/few/small; grade 3, moderate; grade 4, marked/many; grade 5, massive). The grading was applied for each of the predominant kidney lesions like glomerulopathy, and tubular degeneration, atrophy, dilation, and protein casts by a certified pathologist.

### Statistical analysis

For ANG-SD rat study, statistical analysis was performed using one-way ANOVA followed by Dunnett’s multiple comparison versus ANG vehicle-treated rats. *T*-test assuming equivalent variance was used to compare control rats (sham) versus ANG-SD rats. For Ren2TG rat study, statistical analysis was done with Student’s unpaired *t*-test comparisons between vehicle treatment and the respective runcaciguat treatment groups. For comparison of survival rates, the logrank’s test for trend was used. For ZDF rats, statistical analysis was done either with Student’s unpaired *t*-test comparisons between vehicle-treated and runcaciguat-treated rats or by using repeated measures over time, assuming lognormal distribution that has been tested as compared to the Gaussian (a.k.a. normal) distribution. *p < 0.05 versus vehicle. (The respective statistical method is mentioned in the figure legend.)

## Results

### Effects of runcaciguat in Sprague–Dawley rats which were supplemented with angiotensin II (ANG-SD)

The effects of runcaciguat were investigated in a model of hypertension-driven kidney damage with progressive proteinuria. Runcaciguat was administered orally (per oral gavage) for 2 weeks to 12-week-old SD rats that continuously received angiotensin II (ANG, 450 ng/kg/min) delivered by subcutaneous minipumps.

#### Effects of runcaciguat on blood pressure

**Fig. 1:**
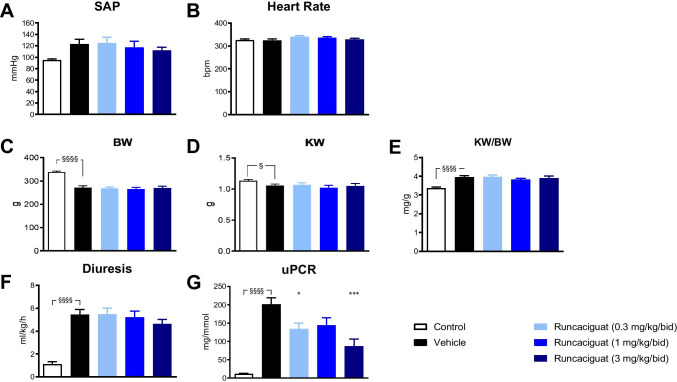
Effects of runcaciguat in ANG-SD rats on **A** systolic arterial pressure (SAP), **B** heart rate, **C** body weight (BW), **D** kidney weight (KW), **E** ratio KW/BW, **F** diuresis, and **G** proteinuria (uPCR) at study end (2 weeks of treatment). ANG-SD rats were treated orally twice a day (bid) per gavage with either vehicle or runcaciguat (0.3, 1.0, and 3.0 mg/kg). Data are mean ± SEM, *N* = 12/group. Significant changes between SD (control) and ANG-SD vehicle-treated rats were determined by *t*-test, §/§§/§§§/§§§§ *p* < 0.05/0.01/0.001/0.0001. Significant changes by treatment of ANG-SD rats were determined by one-way ANOVA followed by Dunnett’s multiple comparison test, */***/**** *P* < 0.05/0.001/0.0001

ANG infusion significantly increased systolic arterial pressure (SAP), from 95.0 ± 1.9 to 122.9 ± 8.6 mmHg (*p* < 0.01) (Fig. 1A) of SD rats without changing heart rate (Fig. 1B). Treatment of ANG-SD rats with runcaciguat (0.3, 1, and 3 mg/kg/bid) did not significantly attenuate the ANG-driven increase of SAP (124.9 ± 10.0 mmHg, 117.4 ± 10.4 mmHg, and 112.2 ± 5.3 mmHg at doses 0.3, 1, and 3 mg/kg/bid, respectively) compared to vehicle-treated rats (122.9 ± 8.6 mmHg) (Fig. 1A) suggesting a blood pressure neutral dosing of the sGC activator. None of the treatments changed heart rate of ANG-SD rats that was approximately 300 bpm as measured at the end of the 2-week treatment (Fig. 1B).

#### Effects of runcaciguat on survival rate

Infusion of ANG to SD rats for 2 weeks did not induce higher mortality rates in the vehicle-treated group. Therefore, a potential beneficial impact of runcaciguat on survival rate of SD-ANG rats could not be assessed under those study conditions.

#### Effects of runcaciguat on body and kidney weights

Infusion of ANG to SD rats for 2 weeks induced a very significant reduction of body weights (by 24%) (Fig. 1C) and a significant reduction in kidney weights (by 7%) (Fig. 1D) compared to SD controls. As a consequence, the ratio kidney weight/body weight (KW/BW) was significantly increased in ANG-SD rats compared to SD controls (by 15%) (Fig. 1E). After the treatment duration of 2 weeks, runcaciguat did not change BW and kidney weight in ANG-SD rats compared to control treatment (Fig. 1C–D, Table 1). Therefore, also, no significant effect on the KW/BW ratio could be observed (Fig. 1D, Table 1).

**Table 1:** Effects of runcaciguat in ANG-supplemented SD rats on body weight (BW), kidney weight (KW), and KW to BW ratios at study end (2 weeks of treatment). ANG-SD rats were treated with either vehicle or runcaciguat (0.3, 1, and 3 mg/kg/bid). Data are mean ± SEM; *N* = 12/group. Significant changes were determined by one-way ANOVA followed by multiple comparison with */**/***/**** for *P* < 0.05/0.01/0.002/0.001/0.0001 compared to control rats

Treatment groups	BW [g]	KW [g]	KW/BW [g/kg]
Vehicle	268,5 ± 7	1.069 ± 0.021***	4.0 ± 0.1
Runcaciguat 0.3 mg/kg/bid	269 ± 5	1.067 ± 0.034***	4.0 ± 0.1
Runcaciguat 1 mg/kg/bid	266 ± 6	1.020 ± 0.039***	3.8 ± 0.1 *P* = 0.051
Runcaciguat 3 mg/kg/bid	270 ± 7	1.053 ± 0.037****	3.9 ± 0.1

#### Effects of runcaciguat on diuresis and proteinuria

Besides the significant blood pressure increase, the infusion of ANG for 2 weeks also increased diuresis by approximately fivefold (Fig. 1F) and proteinuria (uPCR) by approximately tenfold (Fig. 1G) when compared to control SD rats which indicated a hypertension-driven nephropathy. Although runcaciguat at 0.3, 1.0, and 3.0 mg/kg/bid did not change KW/BW at all three doses (Fig. 1D), it significantly reduced proteinuria in ANG-SD rats (at 0.3 and 3 mg/kg/bid) when compared to vehicle (Fig. 1G).

#### Effects of runcaciguat on biomarkers of kidney damage

**Fig. 2:**
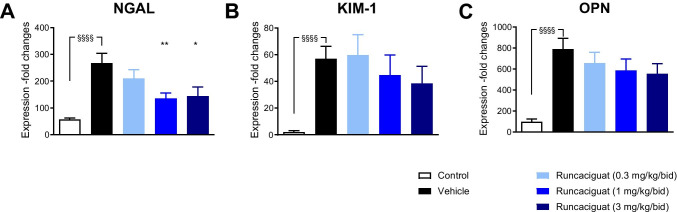
Effects of runcaciguat on renal gene expression in ANG-SD rats treated with either vehicle or runcaciguat (0.3, 1, or 3 mg/kg/bid); change of relative expression of **A** NGAL, **B** KIM-1, and **C** OPN at study end (2 weeks of treatment). Data are mean ± SEM, *N* = 12/group. Significant changes between SD (control) and ANG-SD vehicle-treated rats were determined by *t*-test with §§§§ for *p* < 0.0001. Significant changes by treatment of ANG-SD were determined by one-way ANOVA followed by Dunnett’s multiple comparison test with */**/***/***/ for *P* < 0.05/0.01/0.001/0.0001

Infusion of ANG to SD rats induced dramatic increase in the expression of markers of kidney damage and inflammation, namely of the neutrophil gelatinase-associated lipocalin (NGAL), the kidney injury molecule 1 (KIM-1), and osteopontin (OPN) (Fig. 2A, B, C) compared to SD controls. The reduction of proteinuria induced by runcaciguat in ANG-SD rats was accompanied by a dose-dependent and significant reduction of NGAL gene expression in kidney, a marker of kidney injury (Fig. 2A). Runcaciguat treatment also showed a dose-dependent reduction in the expression of other markers of kidney damage like KIM-1 and OPN (Fig. 2B, C). In summary, the data show that the sGC activator runcaciguat improves kidney function accompanied by a reduction in kidney damage after 2 weeks of treatment of ANG-SD hypertensive rats without reducing blood pressure.

After having demonstrated that runcaciguat can reduce proteinuria in a rat model of hypertension and proteinuria (ANG-SD rats), we investigated its potential cardiovascular and cardio-renal benefits in a more disease-relevant rat model with low nitric oxide (NO) bioavailability, interruption of the NO-sGC signaling, endothelial dysfunction, and hypertension. For these studies, the renin transgenic (RenTG) rat (TGR(mRenR2)27) model was used.

### Effects of runcaciguat in L-NAME-supplemented renin transgenic rats

The RenTG expresses an additional mouse renin gene which renders them severely hypertensive (Mullins et al., 1990, Ganten et al., 1991). In addition, the RenTG rats were chronically supplemented with the NO synthase inhibitor N-nitro-L-arginine methyl ester (L-NAME) for the entire study duration which was applied in the drinking water. L-NAME-supplemented RenTG rats develop malignant hypertension and endothelial dysfunction which results in a significant heart and kidney damage with progressive proteinuria and ultimately in increased mortality rates. In this rat model, the effects of twice daily oral administration of runcaciguat (1, 3, and 10 mg/kg/bid) on cardiac and renal parameters were investigated and compared to vehicle-treated rats.

#### Effects of runcaciguat on blood pressure

**Fig. 3:**
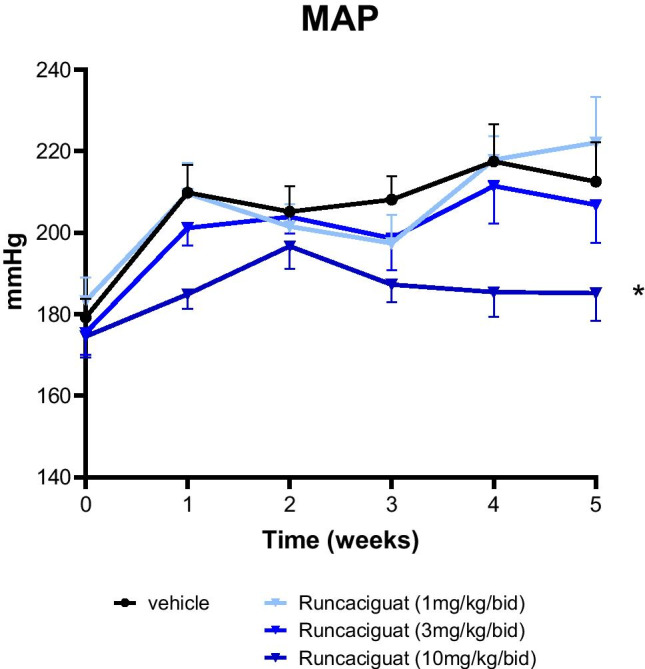
Effects of runcaciguat on mean arterial blood pressure (MAP) of L-NAME-supplemented RenTG rats treated with either vehicle or runcaciguat (0.3, 1, or 3 mg/kg/bid). MAP was measured by non-invasive tail-cuff method. Data are mean ± SEM, *N* = 24/group or *N* = 18/group with vehicle or runcaciguat, respectively. Significant changes were determined by ANOVA with */** *P* < 0.02/0.005

Blood pressure was measured in the conscious hypertensive L-NAME-supplemented RenTG rats via the tail-cuff method before the start of the study (day 0, baseline) and then weekly during treatment period. In vehicle-treated rats (control group), the mean arterial pressure (MAP) increased remarkably from 179.3 ± 4.5 at baseline to 212.9 ± 6.0 mmHg after 7 weeks of treatment (Fig. 3). The increase in MAP could not be blunted or attenuated by runcaciguat. However, the treatment with 10 mg/kg/bid runcaciguat led to a consistent and significant decrease in MAP of about 20 mmHg from weeks 1 to 6 during the study (Fig. 3). This reduction was not observed with the lower doses of 1 and 3 mg/kg/bid runcaciguat (Fig. 3).

#### Effects of runcaciguat on survival rate

**Fig. 4:**
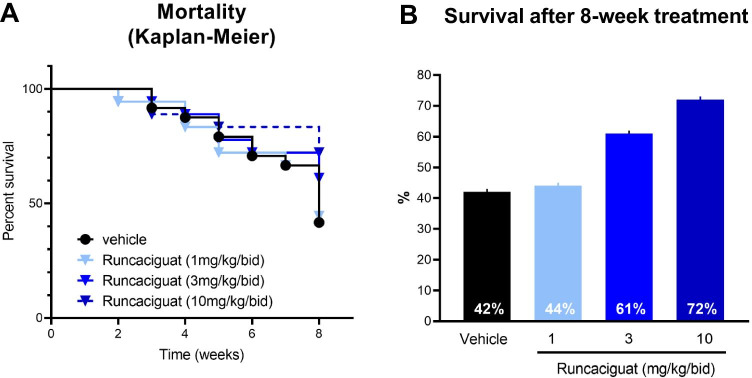
Effects of runcaciguat on **A** survival (Kaplan–Meier plot) of L-NAME-supplemented RenTG rats treated with either vehicle or runcaciguat (0.3, 1, or 3 mg/kg/bid) (left). **B** Survival rates in percent, calculated as percentage of alive rats at study end (8 weeks of treatment) related to group sizes at the beginning of the study (*N* = 24 in vehicle group or *N* = 18 in runcaciguat groups)

It is well established that in the hypertensive L-NAME-supplemented RenTG rats, the mortality rates are high. During the 8-week-treatment duration, 10 out of 24 rats (42%) survived in the vehicle-treated control group (Fig. 4). In contrast, treatment with runcaciguat increased survival rates in a dose-dependent manner, whereas 1 mg/kg runcaciguat did not influence survival significantly (8 out of 18 rats (44%) survived), treatment with runcaciguat at 3 and 10 mg/kg/bid improved survival up to 11 out of 18 rats (61%) and 13 out of 18 rats (72%), respectively (Fig. 4).

#### Effects of runcaciguat on body weight and kidney weight

The dose-dependent improvement of survival of runcaciguat-treated animals was paralleled by an increase in body weight measured after 8 weeks of treatment in runcaciguat (1 mg/kg/bid: 445 ± 16 g (*p* < 0.01), 3 mg/kg/bid: 427 ± 17 g (*p* = 0.05), and 10 mg/kg/bid: 441 ± 13 g (*p* < 0.01)) compared to vehicle (390 ± 12 g) (Table 2; Suppl. Figure 1). Runcaciguat induced a slight and progressive increase in body weight (BW) at each tested dose that reached significance versus vehicle after 8 weeks for groups treated with runcaciguat 1 and 10 mg/kg/bid (Table 2, Suppl. Figure 1). These data suggest a beneficial effect of runcaciguat on overall rat morbidity. In addition, to body weight changes, we evaluated runcaciguat treatment effects on heart and kidney to assess heart and kidney hypertrophy. Therefore, the relative heart weight to body weight ratio and the relative kidney weight to body weight ratio were calculated. Kidney hypertrophy was decreased from 7.48 ± 0.06 g/kg in the control group, compared to 7.12 ± 0.01 g/kg, 7.13 ± 0.11 g/kg, and 7.17 ± 0.09 g/kg in the 1, 3, and 10 mg/kg/bid runcaciguat treatment arm, respectively (Table 2). Interestingly, the relative heart weight was also significantly decreased from 4.02 ± 0.12 g/kg in the control group, compared to 3.5 ± 0.09 g/kg, 3.5 ± 0.09 g/kg, and 3.4 ± 0.06 g/kg in the 1, 3, and 10 mg/kg/bid runcaciguat treatment arm, respectively (Table 2). In particular, the relative left ventricular weight was decreased from 3.40 ± 0.11 g/kg in the control group, compared to 2.94 ± 0.07 g/kg, 3.00 ± 0.08 g/kg, and 2.83 ± 0.05 g/kg in the 1, 3, and 10 mg/kg/bid runcaciguat treatment arm, respectively (Table 2). The relative right ventricular weight was decreased from 0.62 ± 0.03 g/kg in the control group, compared to 0.53 ± 0.02 g/kg, 0.54 ± 0.02 g/kg, and 0.55 ± 0.02 g/kg in the 1, 3, and 10 mg/kg/bid runcaciguat treatment arm, respectively (Table 2). These data suggest an antihypertrophic effect of runcaciguat in the heart.

**Table 2:** Effects of runcaciguat in L-NAME-supplemented RenTG rats on body weight (BW) and organ weight/BW ratios from heart (HW), right and left ventricle (RVW, LVW), and kidney (KW) at study end (8 weeks of treatment). L-NAME-RenTG rats were treated with either vehicle or runcaciguat (0.3, 1, or 3 mg/kg/bid). Data are mean ± SEM; *N* = 8–13/group. Significant changes were determined by one-way ANOVA followed by multiple comparison with */**/***/**** for *P* < 0.05/0.01/0.002/0.001/0.0001

Treatment groups	BW [g]	HW/BW [g/kg]	LVW/BW [g/kg]	RVW/BW [g/kg]	KW/BW [g/kg]
Vehicle	390 ± 12	4.02 ± 0.12	3.40 ± 0.11	0.62 ± 0.03	7.48 ± 0.06
Runcaciguat 1 mg/kg/bid	455 ± 16*	3.47 ± 0.09***	2.94 ± 0.07***	0.53 ± 0.03*	7.12 ± 0.15
Runcaciguat 3 mg/kg/bid	427 ± 17 *P* = 0.050	3.55 ± 0.09***	3.00 ± 0.07**	0.54 ± 0.02 *P* = 0.051	7.13 ± 0.11 *P* = 0.051
Runcaciguat 10 mg/kg/bid	441 ± 13*	3.38 ± 0.06****	2.83 ± 0.05****	0.55 ± 0.01	7.17 ± 0.09

#### Effects of runcaciguat on proteinuria and kidney function

**Fig. 5:**
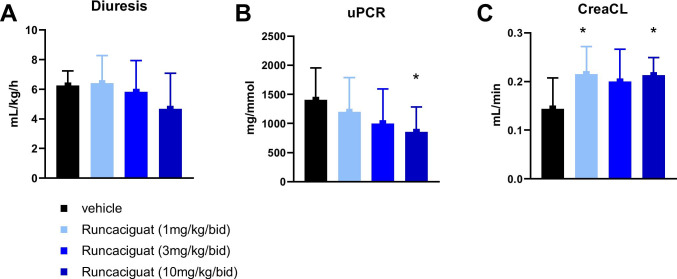
Effects of runcaciguat on **A** diuresis, **B** proteinuria at week 6, and **C** creatinine clearance at study end (8 weeks of treatment) in L-NAME-supplemented RenTG rats treated with either vehicle or runcaciguat (0.3, 1, or 3 mg/kg/bid). Data are mean ± SEM; *N* = 8–13/group. Significant changes were determined by one-way ANOVA followed by Dunnett’s multiple comparison with * for *P* < 0.05

In parallel to the reduction of kidney and heart hypertrophy as well as of mortality after 8 weeks of treatment in L-NAME-supplemented RenTG rats, runcaciguat dose-dependently reduced proteinuria (uPCR) when compared to the vehicle treatment (Fig. 5B). Runcaciguat increased creatinine clearance (CreaCl) to nearly the same extent at all three doses tested (1, 3, and 10 mg/kg/bid) (Fig. 5C) without affecting diuresis (Fig. 5A). Since 1 and 3 mg/kg runcaciguat treatment did not influence blood pressure (Fig. 3A), these data suggest that the beneficial effects of runcaciguat on kidney function are — at least to a great extent — independent of blood pressure lowering.

#### Effects of runcaciguat on biomarkers of kidney damage

**Fig. 6:**
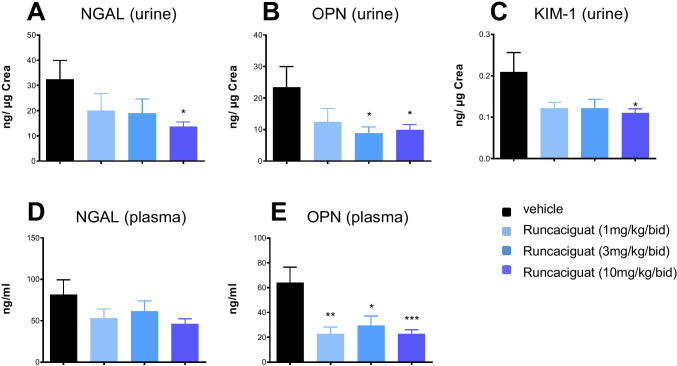
Effects of runcaciguat on urinary and plasma biomarkers in L-NAME-supplemented RenTG rats treated with either vehicle or runcaciguat (0.3, 1, or 3 mg/kg/bid) at study end (8 weeks of treatment). Urinary levels of **A** NGAL, **B** OPN, and **C** KIM-1 and plasma levels of **D** NGAL and **E** OPN. Data are mean ± SEM; *N* = 8–13 per group after completion of the study. Significant changes were determined by one-way ANOVA followed by Dunnett’s multiple comparison with * for *P* < 0.05

The reduction of proteinuria induced by runcaciguat in the L-NAME-supplemented RenTG rats was accompanied by a significant reduction of urinary and plasma biomarkers indicating kidney damage (Fig. 6). In particular, the levels of OPN were reduced at the end of the study (Fig. 6B, E). In addition, NGAL and KIM-1 were reduced in all runcaciguat treatment groups in urine and plasma (Fig. 6A, C, D). However, these effects reached statistical significance only in the highest runcaciguat dose-group in urine (Fig. 6A, C) but not in plasma (Fig. 6D). In line with these results, we also found clearly reduced cystatin C and heart-type fatty acid binding protein (H-FABP) concentrations in both urine and plasma which was also significant in the urine (Suppl. Figure 2). In summary, this reduction of kidney damage biomarkers suggested kidney protection by runcaciguat treatment.

#### Effects of runcaciguat on kidney histopathology

**Fig. 7:**
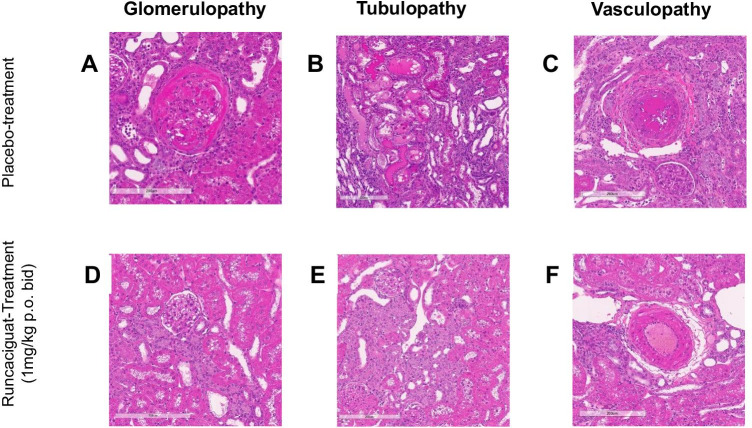
Effects of runcaciguat on renal histopathology in L-NAME-supplemented RenTG rats treated with either vehicle or runcaciguat (0.3, 1, or 3 mg/kg/bid) at study end (8 weeks of treatment). Effects on glomerulopathy (left), tubuls degeneration (middle), and vasculopathy (right) at study end; **A**, **B**, **C** placebo-treated rats and **D**, **E**, **F** rats treated with runcaciguat (1 mg/kg bid) (lower panel). Representative image from *N* = 10/group

To further strengthen and confirm the kidney protective effects indicated by the reduction of biomarkers, morphological and histopathological analyses were performed (Fig. 7). At the end of the study, which was actively terminated after 58% of the placebo-treated animals have died, organs were isolated and prepared for histopathological assessment. Placebo-treated L-NAME-supplemented RenTG rats showed strong degenerative lesions in the kidneys and high severity scores of tubulopathy and vasculopathy (Fig. 7A, B, C). Treatment with runcaciguat dose-dependently reduced kidney damage scores beginning already at the 1 mg/kg/bid dose (Fig. 7D, E, F). Kidney damage was seen in all rats and the mean score in the vehicle-treated group for glomerulopathy was 2.6 which included glomerular dilation and hyaline casts (Fig. 7A), for tubulopathy including not only tubular dilation and tubular degeneration but also hyaline casts was 4.6 (Fig. 7B), and for vasculopathy with fibrinous degeneration and necrosis was 3.7 (Fig. 7C). Runcaciguat in the 1 mg/kg bid reduced these severity scores to 2.3, 3.3, and 2.4 for glomerulopathy, tubulopathy, and vasculopathy, respectively (Fig. 7D, E, F). This improvement in damage scores by runcaciguat was fully in line with the reduction of biomarkers for kidney damage, better renal function as the better survival of the rats when treated with runcaciguat.

In summary, runcaciguat showed significant renal protective effects in two animal models with hypertensive etiology in dosages which do not or only moderately decrease blood pressure.

However, CKD is also driven by risk factors beyond hypertension, such as obesity, diabetes, or dyslipidemia. Therefore, to further characterize the potential of runcaciguat in a representative model beyond hypertensive CKD, a rat model of type 2 diabetes and obesity in association with hypertension, the Zucker diabetic fatty (ZDF) rat model (ZDF-Lebr^fa^-Crl), was employed.

#### Effects of runcaciguat in Zucker diabetic fatty rats

ZDF rats develop a progressive decline in kidney function along aging (Shiota and Printz, 2012) and runcaciguat was administered chronically for 42 weeks via the diet (140 ppm, corresponding to 3 mg/kg/bid, po, gavage) and treatment effects were compared to placebo dietary treatment in ZDF rats.

#### Effects of runcaciguat on blood pressure

**Fig. 8:**
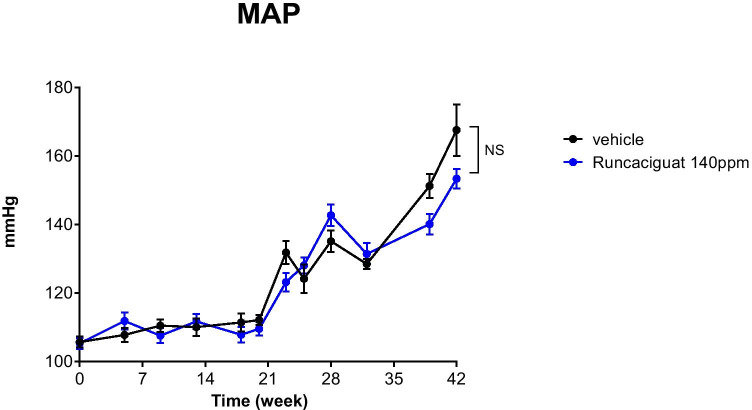
Effects of runcaciguat in conscious ZDF rats treated with either vehicle or runcaciguat (140 ppm) on mean arterial pressure (MAP) measured by tail-cuff at the beginning of the study (week 0) and after 5, 9, 13, 18, 20, 23, 25, 28, 32, 38, and 42 weeks of treatment. Data are mean ± SEM; *N* = 20/group (study start). Significant changes were determined by *t*-test with * for *p* < 0.05

Blood pressure was measured in conscious ZDF rats via the tail-cuff method before the start of the study (day 0) and then regularly every 3–5 weeks up until week 41. Mean arterial pressure (MAP) was not increased until week 20 and increased remarkably and significantly afterwards from 106 ± 2 (at baseline) to 168 ± 8 mmHg in vehicle-treated rats (Fig. 8). The increase in MAP after week 20 till week 41 could not be prevented by runcaciguat treatment (Fig. 8). There was only a moderate and non-significant blood pressure lowering effect observed in runcaciguat treatment group which was 11 mmHg and 15 mmHg at week 38 and week 41, respectively, and all animals irrespective of the runcaciguat treatment remained hypertensive at the end of the study (Fig. 8).

#### Effects of runcaciguat on survival rate

No significant differences in survival were observed between the groups. In the vehicle-treated group, 6 out of 20 animals died within the course of the study (70% survival). In the runcaciguat-treated rats, 5 out of 20 animals died (75% survival) corresponding to an improvement of survival by 5% compared to vehicle-treated rats (data not shown) which was a consistent trend but not as pronounced and not significant when compared to the Ren2TG rat model (Fig. 4).

#### Effects of runcaciguat on body weight and kidney weight

Body weight decreased in the placebo group from 400 ± 6 g from baseline to 324 ± 22 g and in the runcaciguat treatment arm from 400 ± 6 g from baseline to 363 ± 28 g (Table 3). There were no significant differences in organ weight/body weight ratios of kidneys, right ventricles, and the complete hearts although the whole organ weight/body weight ratios decreased numerically. Interestingly, there was a significant decrease for the left ventricular weight to body weight ratio suggesting a reduction of left heart hypertrophy (Table 3). Interestingly, an echocardiography after 39 weeks of treatment showed a significant increase of fractional shortening compared to vehicle-treated group (Suppl. Figure 3).

**Table 3:** Effects of runcaciguat in ZDF rats on body weight (BW) and organ weight/BW ratios from heart (HW), right and left ventricle (RVW, LVW), and kidney (KW) at study end (42 weeks of treatment). ZDF rats were treated with either vehicle or runcaciguat (140 ppm). Data were expressed as mean ± SEM; *N* = 14–15/group. Significant changes were determined with unpaired *t*-test with * for *p* < 0.05

Treatment groups	BW [g]	HW/BW [g/kg]	LVW/BW [g/kg]	RVW/BW [g/kg]	KW/BW [g/kg]
Vehicle	324 ± 21.7	4.37 ± 0.32	3.68 ± 0.27	0.70 ± 0.06	6.99 ± 0.46
Runcaciguat 140 ppm	363 ± 27.8 *p* = 0.161	3.875 ± 0.26 *p* = 0.120	3.12 ± 0.17*	0.76 ± 0.10 *p* = 0.310	6.55 ± 0.67 *p* = 0.318

#### Effects of runcaciguat on proteinuria and kidney function

**Fig. 9:**
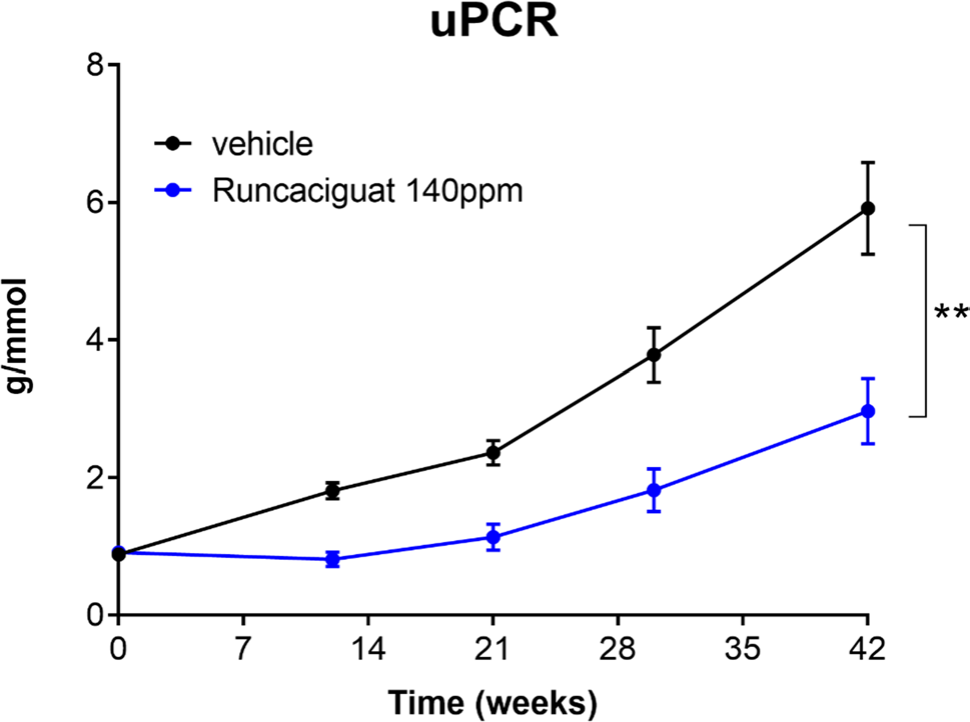
Effects of runcaciguat in conscious ZDF rats treated with either vehicle or runcaciguat (140 ppm) on uPCR at the beginning of the study (week 0) and after 12, 21, 30, and 42 weeks of treatment. Data are mean ± SEM; *N* = 20/group (study start). Significant changes were determined by *t*-test with * for *p* < 0.05

Proteinuria expressed as urinary protein/creatinine ratio (uPCR) was increasing throughout the study reaching a plateau by week 30 (Fig. 9) confirming the severe nephropathy of this diabetic model of CKD. Treatment with runcaciguat significantly attenuated the development of proteinuria (Fig. 9) suggesting a strong renoprotective effect of sGC activator treatment in the ZDF rat model.

#### Effects of runcaciguat on biomarkers of kidney damage

**Fig. 10:**
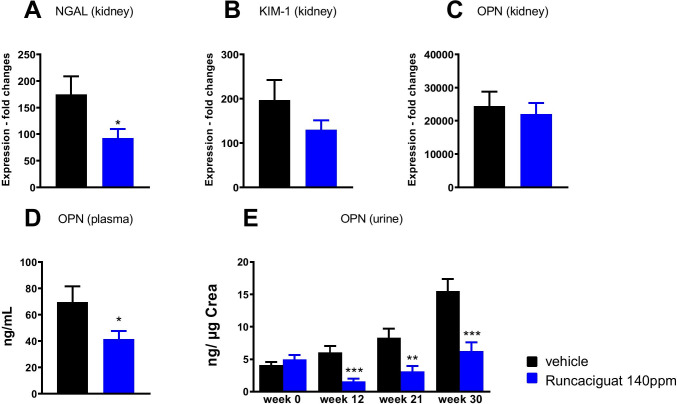
Effects of runcaciguat in ZDF rats treated with either vehicle or runcaciguat (140 ppm) on urinary and plasma biomarkers. Gene expression of **A** NGAL, **B** KIM-1, and **C** OPN in the kidney at study end (42 weeks of treatment). Plasma levels of **D** OPN at study end and **E** urinary OPN levels at baseline (0) and after 12, 21, and 30 weeks of treatment expressed as ratio to urinary creatinine. Data are mean ± SEM; significant changes were determined by one-way ANOVA followed by Dunnett’s multiple comparison or *t*-test with */**/*** for *p* < 0.05/0.01/0.001

Renal gene expression levels as well as plasma and urinary markers of kidney injury were measured to further characterize the nephroprotective capacity of runcaciguat (Fig. 10). Gene expressions of NGAL and KIM-1 were significantly reduced in the kidney after runcaciguat treatment (Fig. 10A, B), whereas the reduction of OPN was only minor and not statistically significant (Fig. 10C). However, runcaciguat significantly reduced plasma OPN concentration at the end of the study (Fig. 10D). In addition, urinary OPN concentrations were significantly and consistently reduced throughout the course of the study (Fig. 10E). Moreover, cystatin C and H-FABP levels in urine were consistently reduced throughout the study (Supple Fig. 4A, B), as were the plasma concentrations of cystatin C and H-FAB at the end of the study (Suppl. Figure 4C, D). In summary, also in the diabetic kidney disease model, not only kidney damage markers but also heart damage markers were reduced by chronic runcaciguat treatment.

#### Effect of runcaciguat on kidney histopathology

**Fig. 11:**
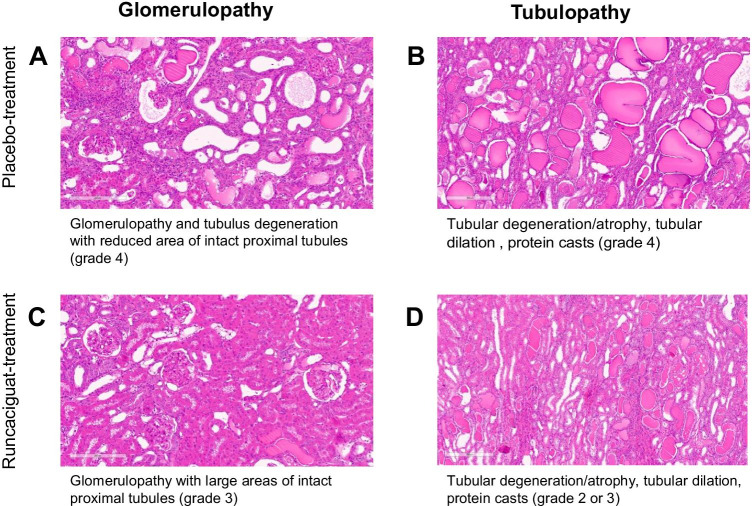
Effects of runcaciguat in ZDF rats treated with either vehicle or runcaciguat (140 ppm) on renal histopathology, glomerulopathy, and tubulus degeneration at study end (42 weeks of treatment); **A**, **B** placebo-treated rats and **C**, **D** rats treated with runcaciguat. Representative images from *N* = 10/group

At the end of the 42 weeks of treatment with runcaciguat, organs were isolated, and histopathological analysis was performed. Glomerulopathy, tubular degeneration/atrophy and dilatation, and protein cast accumulation were evaluated (Fig. 11). In the majority of rats, kidney damage in the vehicle-treated group was severe (grade 4) (Fig. 11A, B). Runcaciguat treatment for 42 weeks led to slight reduction of kidney damage in many rats (grades 2 to 3) with a larger area of intact proximal tubules as compared to vehicle-treated rats (Fig. 11C, D). Overall, this histological observation is in line with the kidney protective effect of runcaciguat as seen for the biomarkers and for the renal function.

In summary, the data generated out of the three different rodent models of CKD and cardio-renal damage showed that runcaciguat reduced proteinuria, improved kidney function, and could reduce mortality. Those effects were already observed not only early after 2 weeks of treatment duration but also chronically after 8 and 42 weeks of treatment and were to a large extent achieved without reduction of systemic blood pressure. These data suggest that the sGC activator runcaciguat induced cardiovascular and cardio-renal protective effects in this preclinical model of CKD associated with diabetes, obesity, and hypertension.

## Discussion

### Could sGC activation provide a new therapeutic approach for chronic kidney disease?

The NO-sGC-cGMP signal transduction pathway is impaired in different cardiovascular diseases, including pulmonary hypertension, heart failure, and chronic kidney diseases (CKDs) (Stasch et al., 2011) Gheorghiade et al., 2013) (Krishnan et al., 2018) (Hofmann, 2020). The impairment of cGMP and NO contributes to sustained hypertension that leads to multi-organ damages especially also in renal tissues (Carlstrom, 2021). However, blood pressure reduction with ACE inhibitors and AT1R antagonists still represents the first-line treatment for CKD patients, and could not fully prevent the progression of renal disease and CKD patients are still requiring more efficacious treatment options. In CKD patients, proteinuria develops concomitantly with hypertension, diabetes, or obesity. These comorbidities result in a pronounced oxidative stress burden, leading to sGC oxidation and therefore disruption of NO binding to the sGC and cGMP signalling. Runcaciguat is a novel NO and heme-independent activator of sGC that can restore cGMP production of sGC activator expressing cells under oxidative stress conditions (Hahn et al., 2021). We therefore, investigated the in vivo effects of runcaciguat in three different CKD models in which proteinuria is driven not only by hypertension but also by diabetes and obesity, namely in angiotensin-treated rats (SD-ANG), in renin transgenic (RenTG) rats supplemented with L-NAME, and in Zucker diabetic fatty (ZDF) rats. In summary, in these different animal models, a dose-dependent kidney protective effect of runcaciguat could be established. Runcaciguat not only reduced proteinuria (Figs. 1, 5, 9) and kidney damage biomarkers (Figs. 2, 6, 10) but also histopathological findings in the kidney (Figs. 7, 11). In RenTG rats, runcaciguat also dose-dependently reduced mortality (Fig. 4).

### Runcaciguat in comparison to other sGC activators in CKD

There is still less information on kidney protective effects of sGC activators published so far (for review, see Stasch et al., 2015). However, it was shown previously that the sGC activator cinaciguat, when given in high doses, could significantly reduce proteinuria and kidney damage in salt-loaded Dahl rats, a model of salt-sensitive hypertension and CKD (Hoffmann et al., 2015). Like earlier studies with sGC activators, the applied dosages significantly reduced blood pressure, which could result in undesired adverse events in patients, and the clinical development of cinaciguat was ultimately stopped due to hypotensive events (Breitenstein et al., 2017). In our studies, and despite the mode of action of sGC activators which NO-independently increase cGMP production and could reduce blood pressure, runcaciguat did not produce significant hypotension nor changes in heart rate in the 3 rodent models. The kidney protective dose (3 mg/kg/bid) which was able to significantly reduce proteinuria (Figs. 1, 5, 9) had no or only a modest influence on blood pressure (Figs. 1, 3, 8). In line with these data, we did not find an activation of the RAAS system in these rats (not shown). More recently, a research group from Boehringer Ingelheim (BI) reported preclinical results with the sGC activator BI 703704 on kidney function in ZSF-1 rats. These rats are characterized by moderate hypertension, T2D, and obesity. BI 703704 demonstrated a dose-dependent kidney protective effect which was also observed in dosages with no or only moderate blood pressure reduction (Boustany-Kari et al., 2016). These kidney protective, but blood pressure neutral effects of BI 703704-treated ZSF-1 rats are fully in line not only with the effects seen with runcaciguat in ZDF rats but also with the effects of runcaciguat in our studies in hypertensive CKD models. Interestingly, runcaciguat and BI 703704 demonstrated in these four different preclinical CKD models a more pronounced reduction of proteinuria in higher, blood pressure lowering dose-regimens, suggesting that there might be also blood pressure-dependent beneficial effects of sGC activators in CKD. Overall, these results seen with sGC activators are fairly comparable from an effect size as from the effects seen on different readouts like uPCR. Therefore, these data suggest a class effect of sGC activators in CKD. It will become really interesting how these highly significant preclinical effects translate in clinical efficacy in CKD patients as currently investigated in a clinical trial with runcaciguat in CKD patients.

### sGC activators and sGC stimulators in CKD

Since early on potent and selective but also orally available sGC stimulators were available, there is also a quite substantial preclinical evidence for a kidney protective effect of sGC stimulators (for review, see Stasch et al., 2015). However, as several preclinical models are driven by arterial hypertension and sGC stimulators were used also in blood pressure reducing dosages, a blood pressure independent, direct kidney protection of sGC stimulators remained to be shown. Therefore, it is very difficult to predict if both principles could be effective in CKD in general or if sGC activators, due to the high oxidative stress burden in CKD, might be even superior. Very recently, a kidney protective effect of the sGC stimulator praliciguat and blood-glucose lowering effects were reported in the ZSF1 rat CKD model (Liu et al., 2020). These effects were at least in part also confirmed in patients with DKD (Hanrahan et al., 2020). Therefore, it will become important in the future to study these effects more systematically and directly compare sGC stimulators and sGC activators not only on blood-glucose lowering but also on different readouts, including metabolic and inflammatory parameters. This will require preclinical head to head comparisons in the future and prior intense dose-finding studies for both mechanisms.

### Mechanisms of action of sGC activators in CKD

The presented data support a substantial kidney protective effect of runcaciguat including a maintenance of kidney function in predictive CKD models with different etiologies. Although these data are coming from predictive in vivo models, these data sets are mostly descriptive. Therefore, one limitation of these studies is that the exact molecular mechanisms of action for these kidney protective effects of runcaciguat could not be elucidated yet. However, it could be demonstrated recently that runcaciguat was able to improve renal blood flow in afferent and efferent arterioles as in isolated perfused kidney preparation (Stehle et al. 2021). These data suggest a better kidney perfusion and oxygenation which could contribute to the kidney protection. In addition, reducing the blood pressure could have some kidney protective effects at least acutely which might be supported by the higher efficacy of blood pressure lowering, e.g., in the hypertensive CKD models. It could not be ruled out that also potential effects on blood glucose may contribute to these effects seen in preclinical models. Treatment with the sGC activator BI 703,704 resulted also in a reduction of blood glucose (HbA1C glucose) in ZSF-1 rats (Boustany-Kari et al. 2016). We also found a decrease of the HbA1C levels by runcaciguat treatment in ZDF-1 rats in pilot experiments (data not shown). Thus, improved glycemic control could also contribute to the mode of action of sGC activators, especially in DKD patients. In the future, other studies, which focus on the molecular mode of action, are required also to identify distinct patient populations which will benefit most from the sGC activator therapy.

### What could be the potential of sGC activators in cardio-renal diseases and heart failure?

It has been described previously that sGC stimulators had beneficial effects in preclinical models of heart failure (Gheorghiade et al., 2013). In fact, the sGC stimulator vericiguat was recently approved for the treatment of HFrEF (Markham and Duggan, 2021). Although sGC stimulators are efficacious in HFrEF, two phase 2 studies in HFpEF patients with the sGC stimulators vericiguat and praliciguat could not show a significant benefit (Armstrong et al., 2020a, Udelson et al., 2020). Since one major comorbidity in HFpEF is T2D which can cause significant oxidative stress, we started to assess the effects of runcaciguat on heart damage in the ZDF rat model. Runcaciguat could not only improve fractional shortening (Suppl. Figure 3) but also significantly reduced the heart-specific fatty acid binding protein (H-FAB) pilot results (Suppl. Figures 2, 4) a sensitive heart damage biomarker. Moreover, in RenTG rats which feature some HFpEF characteristics (Kovacs et al., 2016), runcaciguat was able to decrease mortality rates (Fig. 4). Therefore, future studies are required to focus more on the potential of sGC activators in chronic heart diseases, to fully understand the treatment potential of sGC activators in both CKD and chronic HF. This might be of particularly interest in HF with preserved ejection fraction (HFpEF) with its high oxidative stress burden (Kolijn et al., 2020).

### Summary

**Fig. 12:**
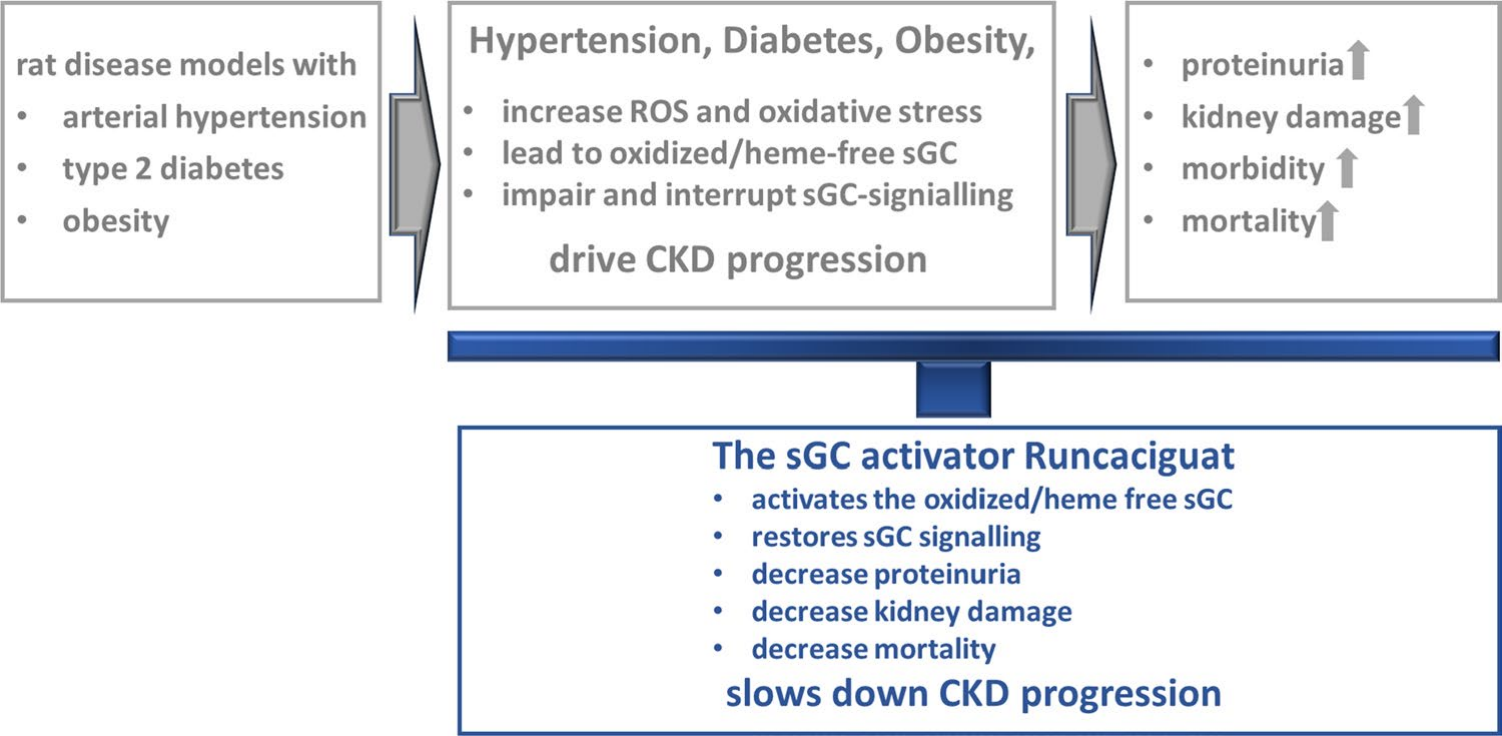
Graphical abstract

In essence, and as summarized in Fig. 12, the sGC activator runcaciguat has shown significant benefits and efficacy in hypertensive and diabetic preclinical CKD models with progressive proteinuria. These data are suggesting a treatment benefit of runcaciguat in patients with diabetic and hypertensive CKD. This significant renal protective effect was also observed in dosages without blood pressure reduction further suggesting a blood pressure-independent mechanism of action in CKD. Consequently, runcaciguat is currently investigated in a phase 2 clinical program in patients with a clinical diagnosis of CKD with diabetes and/or hypertension (CONCORD, NCT04507061).

## Supplementary Information

Below is the link to the electronic supplementary material.

Supplementary file1 Supplement Figure 1: Effects of runcaciguat on body weight (BW) of L-NAME-supplemented RenTG rats treated with either vehicle or runcaciguat (0.3, 1, or 3 mg/kg/bid). Data are mean ± SEM, N=24/group or N=18/group with vehicle or runcaciguat, respectively at study start. Supplement Figure : Effects of runcaciguat on urinary (upper panel) and plasma biomarker (lower panel) in LNAME-supplemented RenTG rats treated with either vehicle or runcaciguat (0.3, 1, or 3mg/kg/bid). Urinary levels of (A) cystatin-C and (B) H-FABP and plasma levels of (C) cystatin-C and (D) H-FABP at study end (8 weeks of treatment). Data are Mean ± SEM; N=8-13/group. Significant changes were determined by one-way ANOVA followed by Dunnett’s multiple comparison with */**/*** for p< 0.05/0.01/0.001. Supplement Figure 3: Effects of runcaciguat in ZDF rats treated with either vehicle or runcaciguat (140 ppm) on heart function (fraction shortening) measured by echocardiography in the conscious rats after 39 weeks of oral treatment. Data are mean ± SEM. N=15/group after 39 weeks. Significant changes were determined by T-test with */**/*** for p< 0.05/0.01/0.001. Supplement Figure 4: Effects of runcaciguat und urinary (upper panel) and plasma biomarkers (lower panel) in ZDF rats treated with either vehicle or runcaciguat (140 ppm). Urinary levels of (A) cystatin-C and (B) H-FABP at baseline (0) and after 12, 21 and 30 weeks of treatment expressed as ratio to urinary creatinine. Plasma levels of (C) cystatin-C and (D) H-FABP at study end (42 weeks of treatment). Data are mean ± SEM; Significant changes were determined by one-way ANOVA followed by Dunnett’s multiple comparison with */**/*** for p<0.05/0.01/ 0.001.(PDF 32 KB)

## Data Availability

All raw data and materials are available and documented on file. All raw data were made available to NASP for peer review to editors and reviewers. The data that support the findings of this study are available from the corresponding author upon reasonable request.

## Abbreviations

ACE: Angiotensin-converting enzyme
ANOVA: Analysis of variance
ANG: Angiotensin II
AT1R: Angiotensin 1 receptor
cGMP: Cyclic guanosine monophosphate
CKD: Chronic kidney disease
bid: Bidaily
BW: Body weight
CTEPH: Chronic thromboembolic pulmonary hypertension
CreaCl: Creatinine clearance
Cyst-C: Cystatin C
ESRD: End-stage renal disease
g: Gram
GRF: Glomerular filtration rate
HF: Heart failure
H-FABP: Heart-type fatty acid binding protein
HFrEF: Heart failure with reduced ejection fraction
HFpEF: Heart failure with preserved ejection fraction
HW: Heart weight
KIM-1: Kidney injury molecule 1
kg: Kilogram
KW: Kidney weight
L-NAME: Nω-nitro-L-arginine methyl ester
LVR: Left ventricle weight
MAP: Mean arterial pressure
mg: Milligram
ml: Milliliter
mmHg: Millimeter mercury
NGAL: Neutrophil gelatinase-associated lipocalin
NO: Nitric oxide
OPN: Osteopontin
PAH: Pulmonary arterial hypertension
PDE: Phosphodiesterase
po: Per os/oral
ppm: Parts per million
qd: Once daily
RAAS: Renin angiotensin aldosterone system
RenTG: Renin transgenic
RVW: Right ventricle weight
SAP: Systolic arterial pressure
SD: Sprague-Dawley rats
SEM: Standard error of the mean
sGC: Soluble guanylyl cyclase
T2D: Type two diabetes
uPCR: Urinary protein-creatinine ratio
ZDF: Zucker diabetic fatty rats
