# Supplementary figures and images for: Runcaciguat, a novel soluble guanylate cyclase activator, shows renoprotection in hypertensive, diabetic, and metabolic preclinical models of chronic kidney disease
Source: Naunyn Schmiedebergs Arch Pharmacol. 2021 Sep 22;394(12):2363–79. doi: 10.1007/s00210-021-02149-4 (PMC8592982; doi:10.1007/s00210-021-02149-4)

Figure 1 Supplement

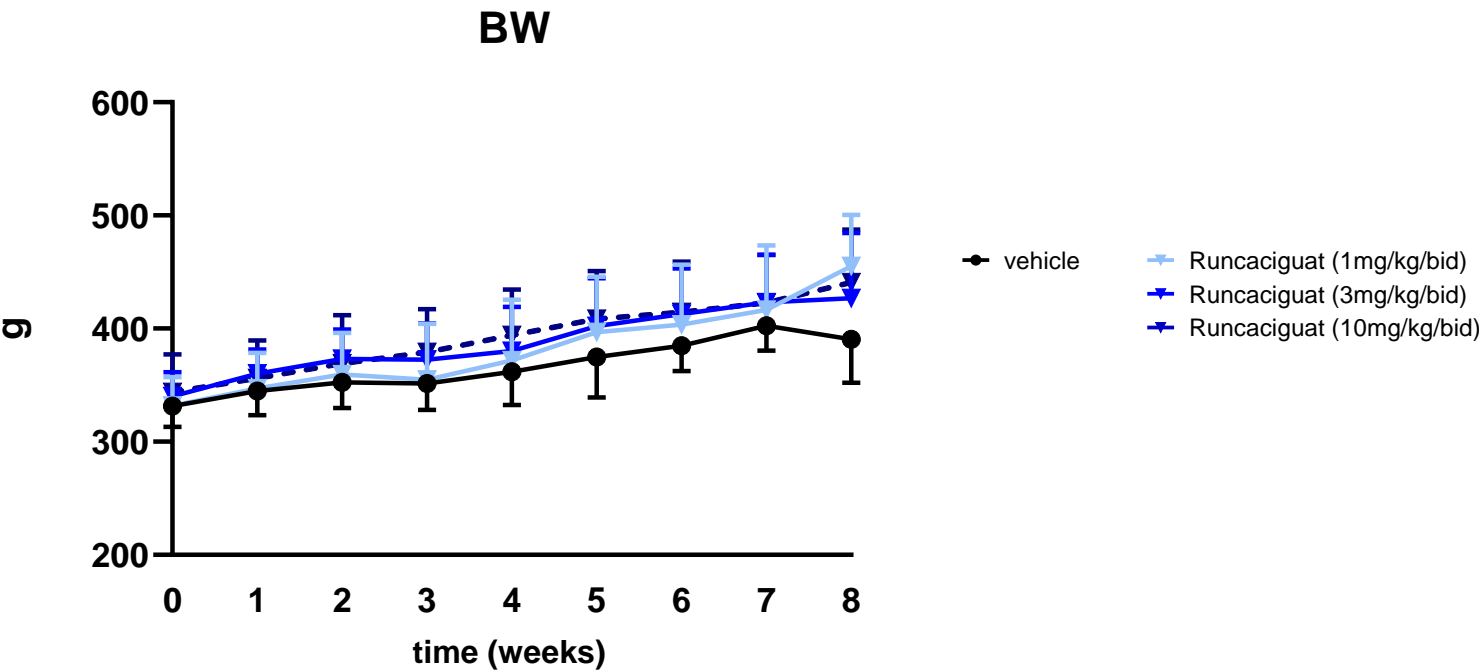

Figure 2 Supplement

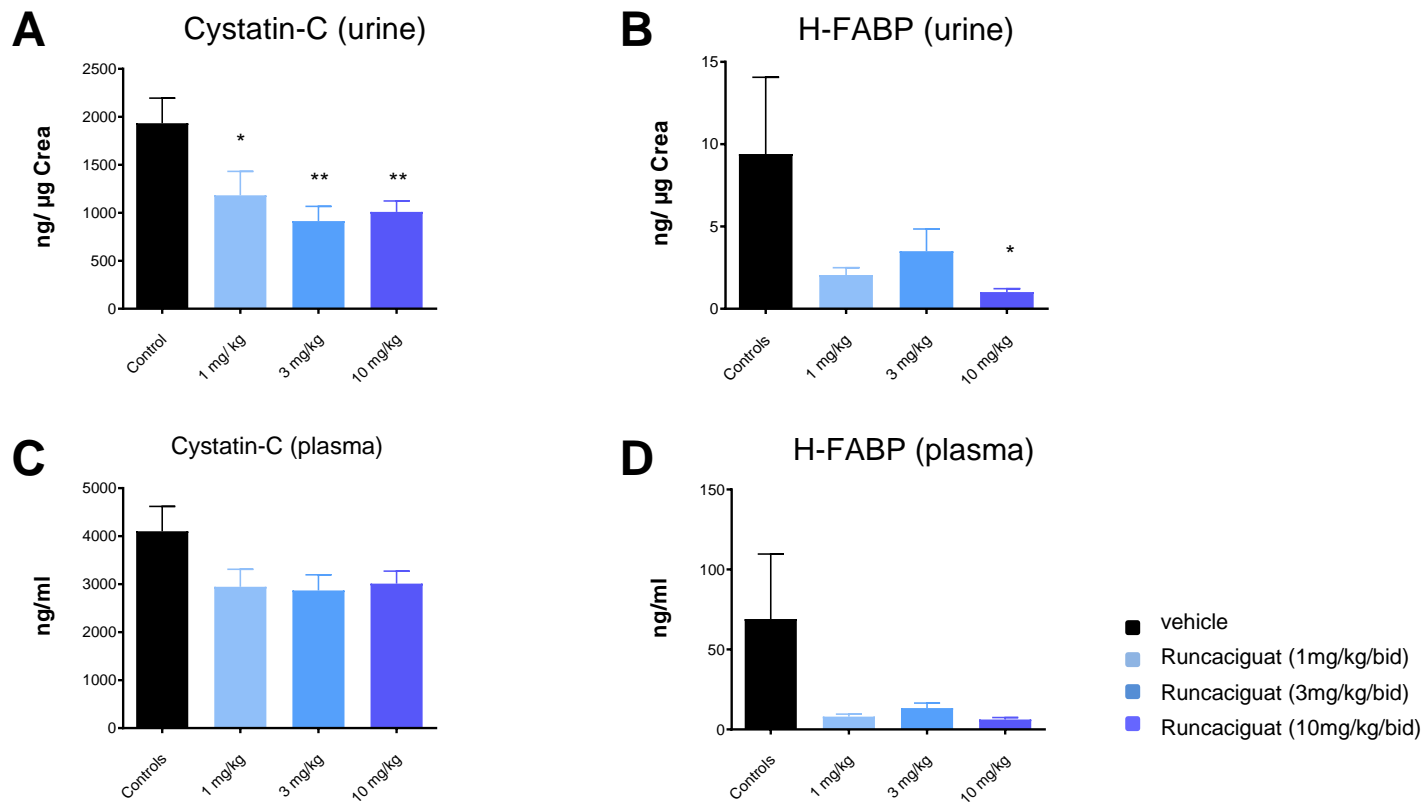

Figure 3 Supplement

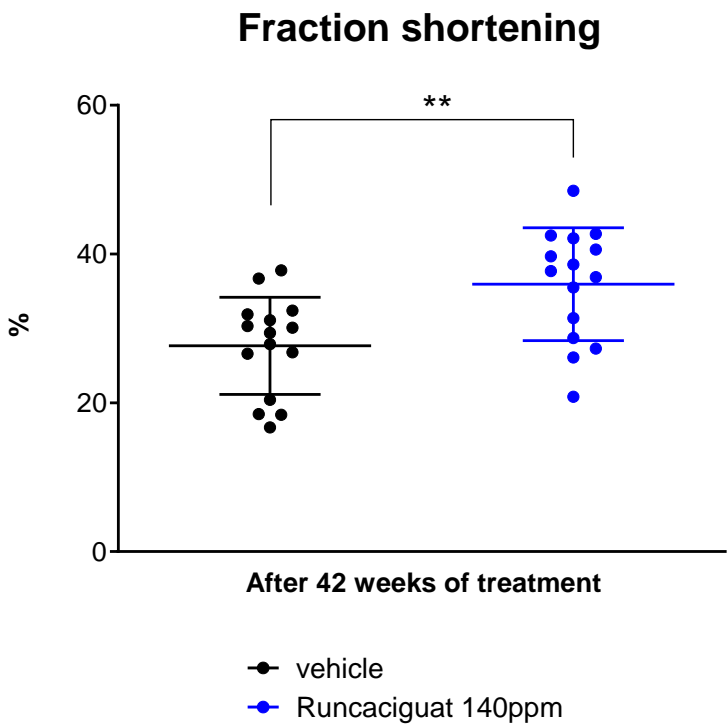

Figure 4 Supplement

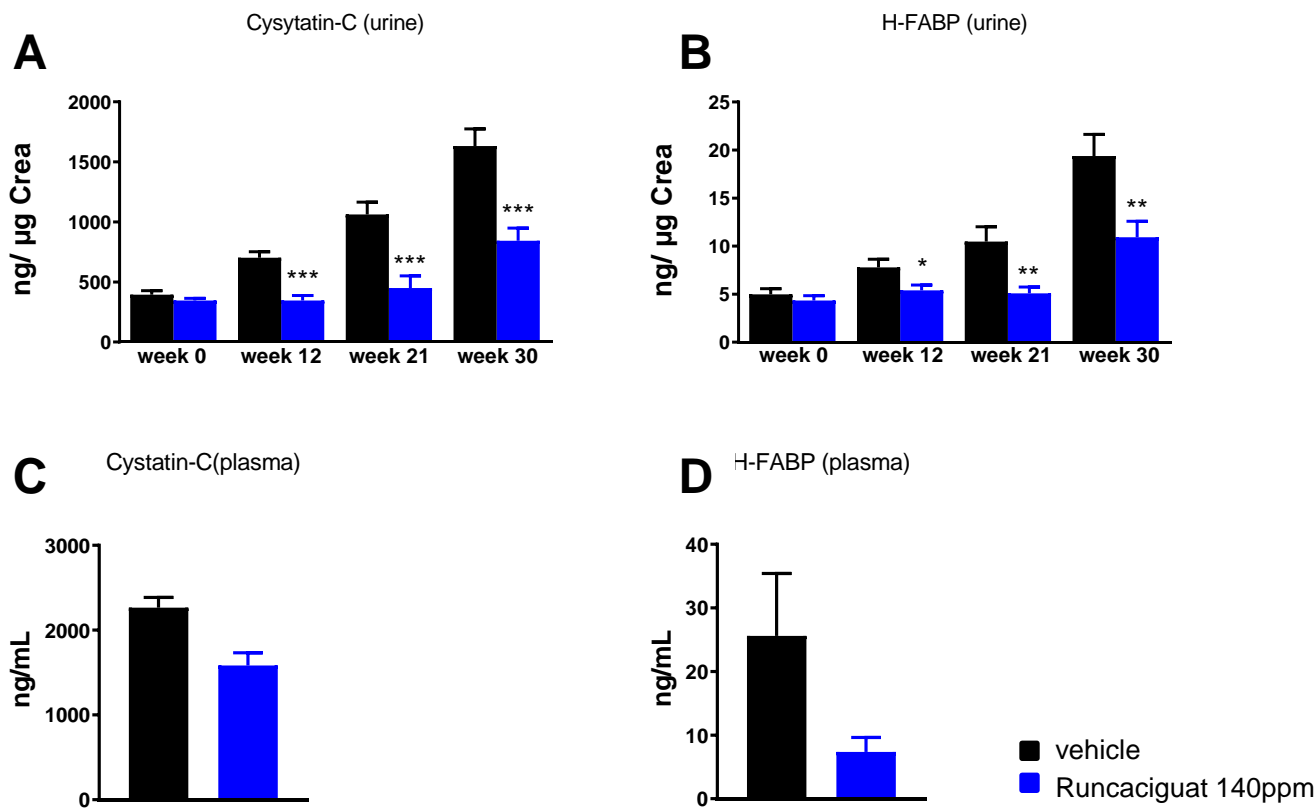

Supplement: Supplementary file 1 — Supplement Figure 1: Effects of runcaciguat on body weight (BW) of L-NAME-supplemented RenTG rats treated with either vehicle or runcaciguat (0.3, 1, or 3 mg/kg/bid). Data are mean ± SEM, N=24/group or N=18/group with vehicle or runcaciguat, respectively at study start. Supplement Figure : Effects of runcaciguat on urinary (upper panel) and plasma biomarker (lower panel) in LNAME-supplemented RenTG rats treated with either vehicle or runcaciguat (0.3, 1, or 3mg/kg/bid). Urinary levels of (A) cystatin-C and (B) H-FABP and plasma levels of (C) cystatin-C and (D) H-FABP at study end (8 weeks of treatment). Data are Mean ± SEM; N=8-13/group. Significant changes were determined by one-way ANOVA followed by Dunnett’s multiple comparison with */**/*** for p< 0.05/0.01/0.001. Supplement Figure 3: Effects of runcaciguat in ZDF rats treated with either vehicle or runcaciguat (140 ppm) on heart function (fraction shortening) measured by echocardiography in the conscious rats after 39 weeks of oral treatment. Data are mean ± SEM. N=15/group after 39 weeks. Significant changes were determined by T-test with */**/*** for p< 0.05/0.01/0.001. Supplement Figure 4: Effects of runcaciguat und urinary (upper panel) and plasma biomarkers (lower panel) in ZDF rats treated with either vehicle or runcaciguat (140 ppm). Urinary levels of (A) cystatin-C and (B) H-FABP at baseline (0) and after 12, 21 and 30 weeks of treatment expressed as ratio to urinary creatinine. Plasma levels of (C) cystatin-C and (D) H-FABP at study end (42 weeks of treatment). Data are mean ± SEM; Significant changes were determined by one-way ANOVA followed by Dunnett’s multiple comparison with */**/*** for p<0.05/0.01/ 0.001.(PDF 32 KB) [file 210_2021_2149_MOESM1_ESM.pdf]
